# Efficacy, Safety, and Tolerability of a Very Low-Calorie Ketogenic Diet in Women with Obesity and Symptomatic Knee Osteoarthritis: A Pilot Interventional Study

**DOI:** 10.3390/nu16193236

**Published:** 2024-09-24

**Authors:** Jacopo Ciaffi, Luana Mancarella, Giulia Pederzani, Lucia Lisi, Veronica Brusi, Federica Pignatti, Susanna Ricci, Giorgia Vitali, Cesare Faldini, Francesco Ursini

**Affiliations:** 1 Medicine & Rheumatology Unit, IRCCS Istituto Ortopedico Rizzoli, 40136 Bologna, Italy; luana.mancarella@ior.it (L.M.); giulia.pederzani2@studio.unibo.it (G.P.); lucia.lisi@ior.it (L.L.); veronica.brusi@ior.it (V.B.); federica.pignatti@ior.it (F.P.); francesco.ursini2@unibo.it (F.U.); 2 Department of Biomedical and Neuromotor Sciences (DIBINEM), University of Bologna, 40127 Bologna, Italy; cesare.faldini@ior.it; 3 Dietetic Service, IRCCS Istituto Ortopedico Rizzoli, 40136 Bologna, Italy; susanna.ricci@ior.it (S.R.); giorgia.vitali@ior.it (G.V.); 4 1st Orthopaedic and Traumatology Department, IRCCS Istituto Ortopedico Rizzoli, 40136 Bologna, Italy

**Keywords:** knee, osteoarthritis, ketogenic diet, ketone bodies, pain, obesity, WOMAC

## Abstract

Background/Objectives: Obesity is a major risk factor for knee osteoarthritis (OA), and weight loss is crucial for its management. This pilot study explores the effects of a Very Low-Calorie Ketogenic Diet (VLCKD) in women with obesity and symptomatic knee OA. Methods: Women with symptomatic knee OA and obesity, defined as a body mass index (BMI) ≥ 30 kg/m^2^, were eligible for the VLCKD protocol. The intervention included a ketogenic phase from baseline (T0) to the 8th week (T8), followed by a progressive reintroduction of carbohydrates over the next 12 weeks, ending at the 20th week (T20). Body mass index (BMI), the Western Ontario and McMaster Universities (WOMAC) Osteoarthritis Index, the EuroQol 5D (EQ-5D), and the 36-item Short Form Health Survey (SF-36) were assessed at all time points. Generalized estimating equations were used to analyze the association between BMI and patient-reported outcomes across the study period. Results: Twenty participants started the study, but four discontinued the intervention, with two of these being due to adverse effects. The mean age of the 16 patients who completed the 20-week program was 57.3 ± 5.5 years, and their mean BMI was 40.0 ± 4.8 kg/m^2^. The mean BMI significantly decreased to 37.5 ± 4.5 at T4, 36.3 ± 4.6 at T8, and 34.8 ± 4.8 at T20 (all *p* < 0.001 compared to baseline). The total WOMAC score improved from a mean of 43.6 ± 16.9 at T0 to 30.2 ± 12.8 at T4 (*p* = 0.005) and further to 24.7 ± 10.6 at T8 (*p* = 0.001) and to 24.8 ± 15.9 at T20 (*p* = 0.005). The reduction in BMI was significantly correlated with the improvements in WOMAC, EQ-5D, and SF-36 over time. No major adverse effects were observed. Conclusions: A 20-week VLCKD in women with obesity and knee OA significantly reduced their weight and improved their outcomes, warranting further research. This trial is registered with number NCT05848544 on ClinicalTrials.gov.

## 1. Introduction

Osteoarthritis (OA) is the most prevalent joint disease, characterized by functional disability, chronic pain, and increasing utilization of healthcare resources [1,2]. The knee is the most frequently affected joint and accounts for 56% of the burden of all sites of OA [3,4]. An estimation from 2020 quantified the prevalence of knee OA in approximately 23% of individuals with an age of 40 years and above, which means roughly 654 million people globally. With a pooled incidence rate of 203 per 10,000 person years among those older than 20 years, nearly 87 million new cases of knee OA are expected each year [5].

For patients with mild to moderate knee OA, or those who are not candidates for arthroplasty, a wide range of treatments has been investigated and used as conservative approaches for symptomatic relief [6]. Commonly used analgesics include acetaminophen, nonsteroidal anti-inflammatory drugs (NSAIDs), duloxetine, and opioids, but their efficacy remains uncertain and they can cause side effects [7,8]. Intra-articular injections of corticosteroids, hyaluronic acid, and platelet-rich plasma are widely used, though the long-term results are inconclusive [9,10,11,12]. Additionally, interventional procedures like genicular artery embolization or the radiofrequency ablation of genicular nerves may be considered [13]. Regenerative approaches, such as stem cell therapy, have also been attempted but with unsatisfactory outcomes [14]. Despite these pharmacological and interventional options, the first-line therapy should always focus on patient education, weight control, and physical exercise, particularly in individuals with obesity [15].

Obesity is a well-known risk factor for knee OA, and the rising prevalence of knee OA is mainly driven by the combined effects of the aging of the population along with the increasing rates of obesity [2,16,17]. The estimated lifetime risk of symptomatic knee OA in individuals with obesity, defined as a body mass index (BMI) ≥ 30 kg/m^2^, is nearly twice as high as in individuals without obesity (20% vs. 11%) [18].

For patients with overweight or obesity and knee OA, weight loss is strongly recommended by all major professional organizations, including the European Alliance of Associations for Rheumatology (EULAR), the American College of Rheumatology (ACR), the Osteoarthritis Research Society International (OARSI) and the American Academy of Orthopedic Surgeons (AAOS) [1,19,20,21].

While diet and exercise are the preferred methods to lose weight, patients may not always be motivated, and even when they are, it can be difficult to achieve and maintain significant weight loss [22]. Weight loss can be achieved through various methods, all of which typically involve a reduction in caloric intake [23]. However, the true challenge lies in maintenance in the long term, as suggested by a meta-analysis of weight loss studies, which found that, in the first two years, over 50% of the lost weight was regained [24].

For this reason, diets that not only restrict calories but also have anti-inflammatory effects have gained interest, and this might also apply to the context of OA, where inflammation is an important factor in the pathophysiology of the disease [25,26]. A Very Low-Calorie Ketogenic Diet (VLCKD) exerts anti-inflammatory and immunomodulatory effects through different mechanisms, including the inhibition of the pro-inflammatory cytokines interleukin-1β, interleukin-6 and tumor necrosis factor α [27,28,29]. Pro-inflammatory cytokines can promote the expression of cartilage-degrading enzymes, such as aggrecanases and metalloproteinases, in chondrocytes, thus altering cartilage homeostasis [30,31].

In rat knee OA models, the high levels of the ketone body β-hydroxybutyrate (βOHB) achieved after 8 weeks of VLCKD, compared to a standard diet, have been shown to significantly inhibit the activation of the NLRP3 inflammasome [32]. This inhibition leads to a reduction in the expression of inflammatory cytokines and metalloproteinases in chondrocytes, as well as an improvement in the microarchitecture of the subchondral bone [32].

Initially developed as a treatment for epilepsy before antiepileptic drugs were available, the concept of inducing ketone body production through a low-carbohydrate diet has evolved to be used in a variety of conditions, from neurodegenerative or oncological diseases to musculoskeletal disorders [33,34,35,36,37]. VLCKD has been particularly useful in promoting weight loss in individuals with obesity who have not responded to other dietary interventions [38]. This success has led to its inclusion in guidelines for the management of obesity [39,40].

Preliminary results suggest that a low-carbohydrate diet may alleviate pain in patients with knee OA, independent of weight loss, although specific evidence for VLCKD remains limited [41]. Since obesity carries an increased risk of knee OA and considering that achieving and maintaining weight loss can be challenging, particularly in women, who have a higher prevalence of OA, investigating the effects of VLCKD in this population is crucial. In this pilot study, focused on women with obesity and symptomatic knee OA, our primary aim is to investigate the efficacy, along with the safety profile and tolerability, of VLCKD as a strategy for reducing the burden of the disease.

## 2. Materials and Methods

### 2.1. Study Design

The trial was a 24-week pilot interventional study conducted at the IRCCS Istituto Ortopedico Rizzoli in Bologna, Italy, from July 2021 to July 2024.

### 2.2. Study Sample

Adult patients with symptomatic knee OA who visited the rheumatology outpatient clinic were considered for inclusion. Bilateral antero-posterior and lateral view X-ray images of both knees were obtained from all patients. The Kellgren–Lawrence grading method was used to quantify the severity of knee OA from doubtful (grade 1) to mild (grade 2), moderate (grade 3), and severe (grade 4) [42]. Due to the exploratory nature of the study, the higher prevalence of the disease among women, and the distinct characteristics between genders, we decided to enroll an arbitrary sample of 20 patients, all of whom were female [43,44].

Patients were deemed eligible for the VLCKD based on the Italian Standards for the Treatment of Obesity, as outlined by the Italian Society for the Study of Obesity and the Italian Association of Dietetics and Clinical Nutrition (2016–2017), as well as the recommendations from the Italian Society of Endocrinology regarding VLCKD [40]. Women aged 18 to 65 years diagnosed with symptomatic knee OA according to the American College of Rheumatology (ACR) criteria were considered for inclusion if they had a BMI of 35.0 kg/m^2^ or higher, or if they had a BMI between 30.0 and 34.9 kg/m^2^ plus at least one additional cardiometabolic risk condition, as detailed in Table 1 [45]. A history of unsuccessful weight loss with standard hypocaloric diets had to be present.

### 2.3. Dietary Intervention

**Figure 1:**
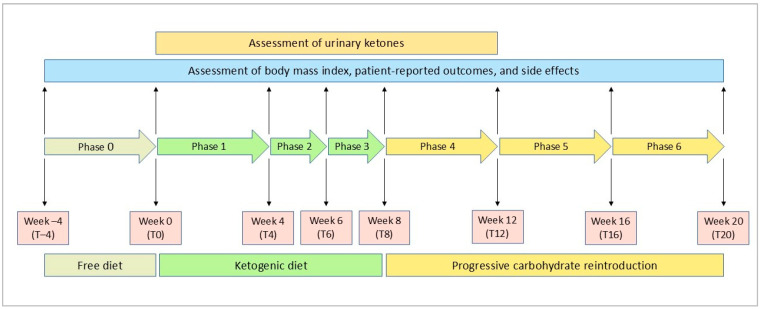
Flowchart of the study protocol.

The study protocol flowchart is illustrated in Figure 1. At the screening visit, a senior rheumatologist and two dietitians met eligible patients to provide information about the practical aspects of VLCKD. Patients also received instructions on the use of a food diary and urine ketone testing strips. Demographic data and a medical history were collected at this stage. A 4-week run-in period preceded the start of the weight loss program. In this free-diet phase, spanning from week −4 to week 0 (phase 0—from T–4 to T0), patients were encouraged to eat their regular meals and maintain a daily food record for two weeks. Personalized diet plans were then developed based on each participant’s preferences, structured as a mix of commercially available ketogenic products and homemade meals. The ketogenic products were supplied by an authorized provider, and one of the dietician investigators distributed them to each patient at the start of the ketogenic phase. The run-in period also served as a self-control phase for the study.

The ketogenic period was structured into three distinct phases. From baseline to the fourth week (phase 1—T0 to T4), patients consumed 4 to 6 protein preparations daily, which were available in various recipes, along with low-carbohydrate vegetables. Each meal preparation provided 90 to 205 kcal, with a protein content between 30% and 72%. Patients were also advised to drink at least 2 L of water or clear liquids (such as tea, coffee, or unsweetened carbonated drinks) per day.

Phase 2 and phase 3 each consisted of two-week periods, spanning from T4 to T6 and T6 to T8, respectively. During these phases, even if the patients were still maintaining ketosis, one meal preparation in phase 2 and two preparations in phase 3 were substituted with natural protein sources such as meat, fish, eggs, or legumes

Following the ketogenic period, carbohydrates were gradually reintroduced over the next 12 weeks, starting with foods of a low glycemic index during the first 4 weeks (phase 4—T8 to T12), then moving to moderate glycemic index foods (phase 5—T12 to T16), and finally including high glycemic index products (phase 6—T16 to T20). This gradual reintroduction aimed to provide nutritional education and reinforce long-term weight loss success.

**Figure 2:**
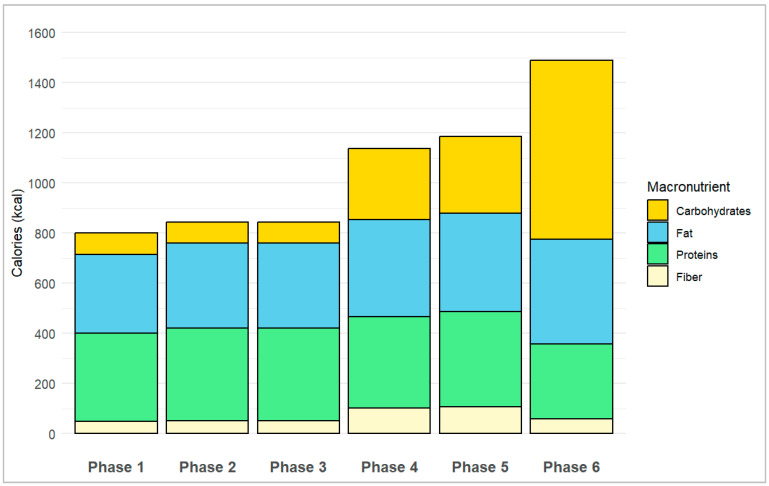
Average daily caloric intake and macronutrients distribution in each study phase.

The average daily caloric intake increased from 801 kcal in the first phase to 843 kcal in the second and third phases, 1138 kcal in the fourth phase, 1186 kcal in the fifth phase, and 1490 kcal in the sixth phase. Regarding the macronutrients distribution, the percentage of proteins decreased from 44% in the first three phases to 32% in the fourth and fifth phases, and 20% in the sixth phase. The percentage of fat changed from 39% (4% saturated) in the first phase to 40% (5% saturated) in the second and third phases, 34% (4% saturated) in the fourth phase, 33% (4% saturated) in the fifth phase, and finally 28% (5% saturated) in the last phase. Conversely, the proportion of carbohydrates increased from 10–11% in the first three phases to 25–26% in the fourth and fifth phases and 48% in the sixth phase. Throughout all the study phases, fiber contributed between 4 and 9% of the daily caloric intake. Figure 2 shows the average daily caloric intake and macronutrient distribution in each study phase.

During the study period, patients were allowed to continue using on-demand analgesic medications. To be eligible for enrolment, at least three months must have passed since their last intra-articular procedure, and no additional procedures were permitted during the study. Patients were also advised to maintain their usual activities without making significant changes, such as altering their physiotherapy, acupuncture, or other non-pharmacological treatments for musculoskeletal pain.

### 2.4. Adherence

Urinary ketosis was monitored using the urine strips provided to each patient at the time of enrolment (Multistix 10SG, Siemens Healthcare Diagnostics, Inc., Tarrytown, NY, USA). Patients were asked to assess ketosis weekly from T0 to T8 and again at T12, and to send a photo of the results to the email address of the study team. During the study period, patients were prohibited from using medications that could potentially interfere with urinary ketone measurements, such as valproic acid, captopril, levodopa, ascorbic acid, or nitrates [46,47,48].

### 2.5. Safety Monitoring

The safety was closely monitored through regular blood and urine tests conducted before the start of the VLCKD, every 4 weeks during the ketogenic phase, and again at the completion of the study. Patients were asked to keep a daily log of their meals and any symptoms of intolerance. Patients were provided with a phone number to report any suspected adverse events, which they or their families could use at any time. Diet tolerance was further assessed during study visits, and adverse events were monitored. In the case of adverse events or intolerance, the patient and the investigators discussed the option to withdraw from the study. A rapid pregnancy test was administered to women of childbearing age before starting the study.

### 2.6. Study Outcomes

Disease activity was assessed using validated patient-reported outcomes (PROs), in line with the OARSI Clinical Trials Recommendations: Design and Conduct of Clinical Trials of Rehabilitation Interventions for Osteoarthritis [49]. These tools included the Western Ontario and McMaster Universities (WOMAC) Osteoarthritis Index, the EuroQoL 5 Dimensions 3 Levels (EQ-5D), and the 36-item Short Form Health Survey (SF-36) [49,50,51,52]. Body weight and PROs were measured every four weeks from T–4 to T20.

#### 2.6.1. Body Mass Index (BMI)

The BMI was calculated using the formula weight (in kg)/height^2^ (m^2^). Body weight was measured at each visit from T–4 to T20, resulting in 7 measurements throughout the study period. Height was measured only at the T–4 visit. Both weight and height were measured using a “Sirio” scale with a height meter, provided by Gima S.p.A., Gessate (Milan), Italy, under the “Gima professional medical products” brand. The instrument has been approved for use in clinical trials at our institution.

#### 2.6.2. Western Ontario and McMaster Universities (WOMAC) Osteoarthritis Index

The WOMAC is a self-administered health status questionnaire comprising 24 items divided into 3 subscales: pain (5 items), stiffness (2 items), and physical function (17 items) [51]. Each item is scored on a Likert scale from 0 to 4. The overall score ranges from 0 to 20 for pain, from 0 to 8 for stiffness, and from 0 to 68 for physical function. The total WOMAC score ranges from 0 to 96. Higher scores correspond to greater levels of pain, stiffness, and functional limitation. The minimal clinically important difference (MCID) for the WOMAC has been reported as 10 for the total WOMAC score following total knee arthroplasty [53]. However, after non-surgical interventions, such as NSAIDs or rehabilitation programs, an MCID of –6.8 has been described [54,55]. Therefore, in our study, we considered a difference of –6.8 in total WOMAC index as clinically meaningful.

#### 2.6.3. EuroQoL-5 Dimensions (EQ-5D)

The EQ-5D is a generic instrument consisting of two distinct parts, and it is widely used to assess quality of life [52]. The first part is a descriptive system with 5 domains: mobility, self-care, usual activities, pain/discomfort, and anxiety/depression. For each domain, patients select one of three severity levels: no problems, some problems, or extreme problems. Responses are then converted into a single utility score using nation-specific weights [56]. In this study, the Italian tariff was applied, with results ranging from −0.39 to 1 [57]. A negative score indicates that patients perceive their health status as worse than death, while a score of 1 represents perfect health. The second part of the questionnaire is a visual analog scale (VAS), where patients rate their current day’s health on a scale from 0 (worst imaginable health) to 100 (best imaginable health).

#### 2.6.4. The 36-Item Short Form Health Survey (SF-36)

The 36-item Short Form Health Survey (SF-36) is a generic multidimensional tool used to assess self-perceived quality of life and health status, focusing on both physical and mental functioning [50]. This tool evaluates eight domains: physical function, physical role, bodily pain, general health, vitality, social function, emotional role, and mental health. Scores range from 0, indicating poor health status, to 100, indicating good health status. In this study, the Italian version of the questionnaire was used [58]. The results were summarized into two overall scores: the physical component score (PCS) and the mental component score (MCS).

### 2.7. Statistical Analysis

Data were expressed as mean ± standard deviation or number (percentage), as appropriate. A paired samples Student’s t-test was used to compare differences in BMI, WOMAC, EQ-5D, and SF-36 between T0 and the various study time points. The longitudinal association between BMI and PROs was analyzed using generalized estimating equation (GEE) models with a linear response and autoregressive correlation structure. All independent models were adjusted for age, with the WOMAC total score, WOMAC pain, WOMAC stiffness, WOMAC function, EQ-5D utility score or VAS, SF-36 MCS, or SF-36 PCS used as the dependent variables. The data were analyzed with a per-protocol approach, including only those patients who achieved ketosis and completed the 20 weeks of the study. A *p*-value < 0.05 was considered statistically significant. The statistical analysis was performed using R Statistical Software, (version 4.4.0; R Foundation for Statistical Computing, Vienna, Austria), and the plots were created using the ‘ggplot2’ package version 3.5.1. [59].

### 2.8. Ethical Considerations

The research was conducted in accordance with the Declaration of Helsinki and its most recent amendments [60]. The study protocol received approval from the local Ethics Committee (Comitato Etico Area Vasta Emilia Centrale, Bologna, Italy—approval number: 0017502/2021), and written informed consent was obtained from all participants. This trial is registered on ClinicalTrials.gov under the number NCT05848544.

## 3. Results

### 3.1. Characteristics of Patients

**Figure 3:**
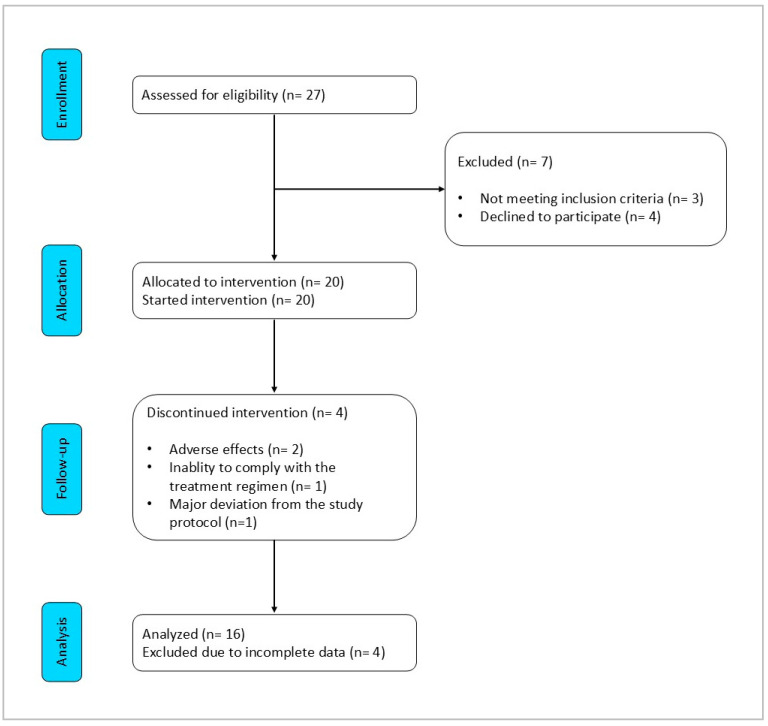
CONSORT flow diagram.

Eligibility was assessed in 27 patients to reach the intended enrolment of 20 participants. Of these, 16 (80%) successfully completed both the 8-week VLCKD phase and the subsequent 12-week carbohydrate reintroduction phase. The Consolidated Standards of Reporting Trials (CONSORT) flow diagram is shown in Figure 3. The characteristics of these patients are summarized in Table 2. The mean age was 57.3 ± 5.5 years, with a mean BMI of 40.0 ± 4.8 kg/m^2^, and 13 patients (81%) had a BMI over 35 kg/m^2^. Among the comorbidities, hypertension (*n* = 9, 56%) and hyperlipidemia (*n* = 8, 50%) were the most prevalent, followed by non-alcoholic fatty liver disease (*n* = 5, 31%). One patient (6%) had diabetes. At baseline, nine patients (56%) reported using paracetamol on demand, while six (38%) used NSAIDs. Assessing the severity of knee OA according to the Kellgren–Lawrence approach, four patients had monolateral grade 2 OA, nine patients had bilateral grade 2 OA, two patients had monolateral grade 3 OA, and one patient had bilateral grade 3 OA.

### 3.2. Adherence to VLCKD

Urine ketone assessments confirmed that all patients achieved ketosis during the 8 weeks of the ketogenic phase, though the degree and consistency varied among individuals. One patient had five positive determinations (6%), six patients had six positive determinations (38%), four patients had seven positive determinations (25%), and five patients had eight positive determinations (31%) (Table 3). Additionally, three participants continued to have detectable urinary ketones at T12.

### 3.3. Safety

The VLCKD was generally well tolerated, but 4 of the 20 enrolled patients (20%) did not complete the study. Two of these dropouts were due to adverse events. One patient discontinued after the first week of the ketogenic phase due to diarrhea and fatigue, while another patient withdrew during the third week due to symptomatic hypotension and dizziness. Of the other two patients, one sustained a lower limb traumatic injury near the end of the sixth week, requiring surgical intervention and thus preventing continuation of the dietary regimen during the rehabilitation period. The last patient was withdrawn from the trial after receiving a corticosteroid knee joint injection at the end of the ketogenic phase.

Among the 16 participants who completed the 20-week intervention, only mild and transient adverse events were reported, occurring in 11 patients (69%). The most common adverse event was constipation (*n* = 6, 38%), followed by fatigue (*n* = 5, 31%). Additionally, three patients (19%) reported headaches, two (13%) experienced diarrhea, and two (13%) had abdominal discomfort. Laboratory tests conducted during the dietary intervention or at the end of the study did not reveal any safety issues.

### 3.4. Efficacy

#### 3.4.1. Change in BMI

**Figure 4:**
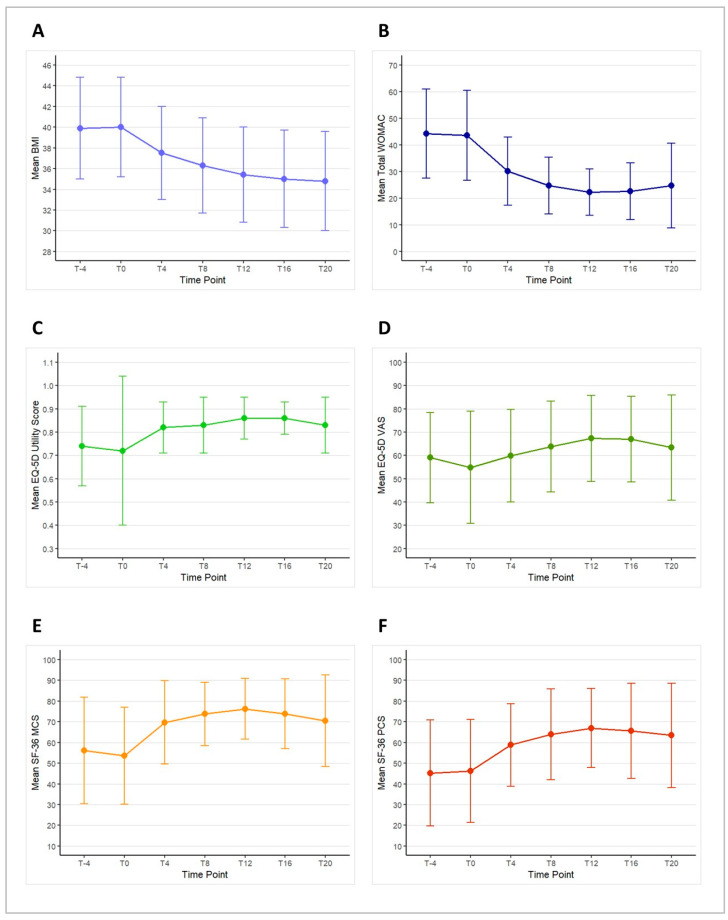
Mean scores and standard deviations of body mass index (BMI—(panel **A**)), Western Ontario and McMaster Universities Osteoarthritis Index (WOMAC—(panel **B**)), the EuroQoL 5 Dimensions 3 Levels (EQ-5D) utility score (panel **C**) and visual analog scale (VAS—(panel **D**)), the 36-item Short Form Health Survey (SF-36) mental component score (MCS—(panel **E**)), and physical component score (PCS—(panel **F**)) across the study time points.

The change in mean BMI from T–4 to T20 is illustrated in Figure 4A. There was no difference in BMI from T–4 to T0, with a mean BMI of 39.9 ± 4.9 at T–4 and 40.0 ± 4.8 at T0 (*p* = 0.358). During the ketogenic phase, there was a significant reduction in mean BMI, dropping to 37.5 ± 4.5 at T4 (*p* < 0.001) and further to 36.3 ± 4.6 at T8 (*p* < 0.001). This decrease in mean BMI remained significant throughout the carbohydrate reintroduction phase, with values of 35.4 ± 4.6 at T12 (*p* < 0.001), 35.0 ± 4.7 at T16 (*p* < 0.001), and 34.8 ± 4.8 at T20 (*p* < 0.001) compared to baseline.

#### 3.4.2. Change in Total WOMAC

The change in mean total WOMAC score from T–4 to T20 is depicted in Figure 4B. During the run-in period, there was no significant change in the mean total WOMAC score, with values of 44.2 ± 16.7 at T–4 and 43.6 ± 16.9 at T0 (*p* = 0.124). During the ketogenic phase, the mean total WOMAC score showed a significant improvement, decreasing to 30.2 ± 12.8 at T4 (*p* = 0.005) and further to 24.7 ± 10.6 at T8 (*p* = 0.001). This positive trend continued into the carbohydrate reintroduction phase, with scores of 22.2 ± 8.7 at T12 (*p* < 0.001), 22.6 ± 10.7 at T16 (*p* = 0.001), and 24.8 ± 15.9 at T20 (*p* = 0.005), all showing significant improvements compared to T0.

When analyzing the progression of total WOMAC scores in individual patients during the VLCKD phase, improvement was observed in 12 patients (75%) at T4, in 14 patients (88%) at T8, and in all 16 patients (100%) at T12. This improvement exceeded the MCID in 9 patients (56%) at T4, 11 patients (69%) at T8, and 12 patients (75%) at T12. Notably, no patient experienced a worsening in total WOMAC that exceeded the MCID. By the end of the study, a clinically meaningful improvement in total WOMAC was still observed in 14 patients (88%).

#### 3.4.3. Change in Each WOMAC Domain

During the free-diet run-in period, no significant change in WOMAC pain was observed, with mean scores of 8.7 ± 3.8 at T–4 and 8.0 ± 3.1 at T0 (*p* = 0.633). Compared to T0, WOMAC pain scores significantly improved during the ketogenic phase, decreasing to 5.6 ± 3.0 at T4 (*p* = 0.015) and further to 4.5 ± 2.4 at T8 (*p* = 0.004). This improvement continued during the carbohydrate reintroduction phase, with WOMAC pain scores dropping to 4.1 ± 2.1 at T12 (*p* = 0.001), 4.3 ± 2.7 at T16 (*p* = 0.003), and 4.2 ± 3.7 at T20 (*p* = 0.005).

For WOMAC stiffness, the mean score was 4.8 ± 1.7 at T–4 and 4.2 ± 1.9 at T0 (*p* = 0.010). Significant improvements were observed compared to T0, with WOMAC stiffness scores decreasing to 2.7 ± 1.7 at T4 (*p* = 0.002) and 2.3 ± 1.5 at T8 (*p* = 0.002). This trend continued, with scores further reducing to 1.9 ± 1.1 at T12 (*p* < 0.001), 1.9 ± 1.2 at T16 (*p* < 0.001), and 2.4 ± 1.7 at T20 (*p* = 0.028).

No significant difference was noted in WOMAC function during the run-in period, with mean scores of 30.7 ± 12.9 at T–4 and 31.3 ± 13.0 at T0 (*p* = 0.568). However, significant improvements in WOMAC function were observed during the ketogenic phase, with scores decreasing to 21.8 ± 9.7 at T4 (*p* = 0.006) and 17.9 ± 8.3 at T8 (*p* = 0.002). The improvement persisted during the carbohydrate reintroduction phase, with WOMAC function scores dropping to 16.2 ± 6.6 at T12 (*p* < 0.001), 16.4 ± 8.0 at T16 (*p* = 0.002), and 18.1 ± 11.5 at T20 (*p* = 0.005).

#### 3.4.4. Change in EQ-5D

Changes in the mean EQ-5D utility score and VAS from T–4 to T20 are presented in Figure 4C,D. During the free-diet run-in period, no significant changes were observed in either the EQ-5D utility score or the VAS score. The mean EQ-5D utility score was 0.74 ± 0.17 at T–4 and 0.72 ± 0.32 at T0 (*p* = 0.772). Although there was an increase in the EQ-5D utility score during the study, it was not statistically significant, with values rising to 0.82 ± 0.11 at T4 (*p* = 0.252), 0.83 ± 0.12 at T8 (*p* = 0.208), 0.86 ± 0.09 at T12 (*p* = 0.125), 0.86 ± 0.07 at T16 (*p* = 0.114), and 0.83 ± 0.12 at T20 (*p* = 0.211).

Similarly, the mean EQ-5D VAS score showed no significant changes during the run-in period, with scores of 59.1 ± 19.4 at T–4 and 54.9 ± 24.0 at T0 (*p* = 0.297). While the VAS score did show some improvement compared to T0, these changes were not statistically significant, with scores of 59.9 ± 19.9 at T4 (*p* = 0.285), 63.8 ± 19.4 at T8 (*p* = 0.187), 67.3 ± 18.5 at T12 (*p* = 0.080), 67.0 ± 18.3 at T16 (*p* = 0.103), and 63.4 ± 22.6 at T20 (*p* = 0.282).

#### 3.4.5. Change in SF-36

Changes in mean SF-36 MCS and PCS from T–4 to T20 are shown in Figure 4E,F. No significant differences were observed in the mean SF-36 MCS or PCS scores between T–4 and T0 during the free-diet run-in period. The mean SF-36 MCS score was 56.1 ± 25.7 at T–4 and 53.6 ± 23.7 at T0 (*p* = 0.329). However, compared to T0, the mean SF-36 MCS score significantly increased to 69.7 ± 20.1 at T4 (*p* = 0.012), 73.8 ± 15.3 at T8 (*p* = 0.004), 76.2 ± 14.7 at T12 (*p* = 0.003), 73.8 ± 16.9 at T16 (*p* = 0.006), and 70.5 ± 22.1 at T20 (*p* = 0.027).

Similarly, the mean SF-36 PCS score was 45.2 ± 25.6 at T–4 and 46.2 ± 24.8 at T0 (*p* = 0.550). Compared to T0, the mean SF-36 PCS score showed a significant improvement, increasing to 58.8 ± 19.9 at T4 (*p* = 0.017), 63.9 ± 22.0 at T8 (*p* = 0.009), 66.9 ± 19.1 at T12 (*p* = 0.003), 65.6 ± 23.0 at T16 (*p* = 0.006), and 63.4 ± 25.2 at T20 (*p* = 0.015).

#### 3.4.6. Association over Time between BMI and Patient-Reported Outcomes

All visits between T–4 and T20 for the 16 patients who completed the study were included in each GEE model, resulting in a total of 112 measurements. The GEE models (Table 4) revealed a significant association between progressive BMI reduction and the longitudinal improvement in the total WOMAC index, WOMAC stiffness, WOMAC function, EQ-5D VAS, and SF-36 PCS. The β coefficients indicate that for each one-point decrease in BMI, a patient is expected to experience a decrease of 1.43 points in the total WOMAC index (95% CI 0.29 to 2.58; *p* = 0.014), a decrease of 0.12 points in WOMAC stiffness (95% CI 0.00 to 0.23; *p* = 0.044), and a decrease of 1.07 points in WOMAC function (95% CI 0.26 to 1.89; *p* = 0.010). Additionally, a one-point decrease in BMI is associated with an increase of 1.74 points in EQ-5D VAS (95% CI 0.39 to 3.09; *p* = 0.012) and an increase of 3.25 points in SF-36 PCS (95% CI 1.84 to 4.65; *p* < 0.001). No association was found between BMI and WOMAC pain or the EQ-5D utility score or SF-36 MCS over time.

## 4. Discussion

We conducted a pilot study to evaluate the efficacy, safety, and tolerability of a VLCKD in patients with obesity and symptomatic knee OA. Our findings suggest that VLCKD has a rapid and positive effect on various aspects of the disease.

We observed an average improvement in total WOMAC of 21.4 points after 12 weeks and 18.8 points by the end of the 20-week study. Although direct comparisons with other treatments are not possible, and our study was not designed for such comparisons, it is important to contextualize the improvement in WOMAC scores with VLCKD by considering how WOMAC scores change following other interventions for knee OA. In a study analyzing the outcomes of 377 total knee arthroplasties (TKAs) one year post-surgery, Liebensteiner et al. reported a significant improvement in median WOMAC scores, from 52 to 10 in patients with advanced OA and from 53 to 19 in those with less severe radiographic disease [61]. Similarly, Leung et al. examined data from 1136 TKA patients and noted a mean total WOMAC improvement from 28.4 before surgery to 7.6 at 6 months, further reducing to 5.2 at 12 months [62].

Switching the attention to minimally invasive interventional procedures, a systematic review and meta-analysis of randomized clinical trials investigating the efficacy of radiofrequency ablation for knee OA showed a mean improvement in WOMAC of 28.4 points at 3 months and of 23.7 points at 6 months [13]. Wang et al. evaluated the effects of intra-articular injections of hyaluronic acid and corticosteroids in 60 knee OA patients, finding that the total WOMAC score improved from a mean of 41 at baseline to 22.7 at 3 months, although it worsened to 39.0 by 6 months [63]. In another study, Smith et al. reported a reduction in total WOMAC scores from 37.5 to 24.0 following a 12-week rehabilitation program [64].

Previous studies on low-energy and very-low-energy diets have already demonstrated their efficacy in achieving a significant weight reduction and improving knee OA symptoms, as measured by the Knee injury and Osteoarthritis Outcome Score (KOOS) and the proportion of patients fulfilling the OMERACT-OARSI responder criteria after 16 weeks of intervention [65]. Besides strict diet regimens, the Mediterranean diet can also play a role in improving OA symptoms. In a large longitudinal study, Veronese et al. showed that knee OA patients who adhered more closely to a Mediterranean diet had a reduced risk of pain worsening [66]. Additionally, a network meta-analysis comparing various weight loss treatments concluded that bariatric surgery, low-calorie diets, or intensive weight loss programs combined with exercise are the most effective in reducing WOMAC pain scores in knee OA patients [67]. The reduction in WOMAC pain was 63% for bariatric surgery, 34% for a low-calorie diet plus exercise, 27% for an intensive weight loss program plus exercise, and 25% for a very low-calorie diet alone. In all cases, the improvement in WOMAC pain was associated with significant weight loss.

Messier et al. analyzed 414 knee OA patients undergoing an 18-month diet and exercise regimen, finding improvements in WOMAC pain from 7.4 to 5.0 and WOMAC function from 25.5 to 16.6 [22]. In our study, we observed consistent improvements, albeit over a substantially shorter period. Mean WOMAC pain scores improved from 8.7 at baseline to 4.2 at week 20, while mean WOMAC function scores improved from 30.7 to 18.1. Although direct comparisons with other studies were not the focus, the improvements in WOMAC observed in our trial highlight the clearly beneficial impact of VLCKD. This suggests that VLCKD is a valid and effective non-invasive treatment option for patients with knee OA.

Furthermore, adherence was high, with 80% of patients completing the trial period. Only two patients discontinued the intervention due to adverse events. It is important to note that following a VLCKD can be challenging for patients. Personalizing each diet plan according to the preferences of each participant, along with constant supervision by a multidisciplinary medical team and close counseling from senior rheumatologists, likely contributed to the high adherence observed.

All patients experienced weight loss, with a significant reduction in BMI observed as early as 4 weeks and continuing throughout the entire study period. Unlike our previous research on VLCKD in patients with fibromyalgia, where improvements in PROs were less directly linked to weight loss, in knee OA, the improvement in PROs was strongly associated with this weight reduction [37].

The anti-inflammatory properties of the ketogenic diet are well documented, and preclinical models of OA have shown that β-hydroxybutyrate, the major ketone body, can exert anti-inflammatory effects on knee articular tissues by inhibiting the NLRP3 inflammasome, thereby reducing bone and cartilage damage [32,68,69]. In our study, the improvements in pain and functional outcomes appear to be predominantly mediated by weight loss. However, our findings do not allow us to fully distinguish between the mechanical benefits of weight reduction and the potential anti-inflammatory effects of VLCKD on PROs. Further research is needed to determine whether the benefits of the ketogenic diet on musculoskeletal pain extend beyond those attributable to weight loss alone.

Our study has several strengths. First, it demonstrates that patients with obesity and knee OA can be sufficiently motivated to engage in challenging and restrictive nutritional protocols aimed at improving their health. Second, a personalized diet plan, which included a variety of meal options and simple recipes tailored to individual preferences, contributed to high adherence and sustained ketosis in most participants. Third, we minimized assessment bias by using validated tools to evaluate various disease domains, including pain, stiffness, physical function, mental distress, and quality of life.

The findings of our pilot study are encouraging, but several limitations should be acknowledged. The small sample size, the inclusion of only women, and the relatively short duration of the intervention may limit the generalizability of our results, particularly to male patients and those with varying severities of knee OA. These factors also prevented us from conducting post hoc subgroup analyses on patients with different disease characteristics, such as a varying OA severity or differing levels of ketosis during the early weeks of the study. Such analyses could have helped distinguish the benefits of weight reduction from those potentially attributed to ketosis. The intervention was unblinded, and this is particularly important to note, as studies on investigational treatments for knee OA often report a significant placebo response, which can impede the ability to meet study endpoints [70]. This effect is more pronounced in trials involving intra-articular treatments, which typically show a larger placebo effect compared to topical or oral placebos [71,72]. Zhang W. found that in OA patients, the percentage of improvement attributed to placebo can be as high as 75% for pain reduction, 71% for functional improvement, and 83% for reduced stiffness [73].

Oral placebo administration has also been shown to improve WOMAC scores, as outlined in a recent meta-analysis by Wen et al. This study suggests that the “efficacy plateau”, that is the time needed to reach 90% of maximum efficacy, occurs between 5 and 7 weeks [74]. Therefore, to adequately evaluate the effectiveness of any intervention in OA, study durations should extend beyond 8 weeks, as we did in our trial.

The high placebo response in OA clinical trials often complicates the detection of a potentially effective drug, with variability depending on the type of placebo used [75]. Although the placebo effect could not be directly assessed in our study, we believe it does not impair the interpretability of our results. We implemented a self-controlled design to mitigate this. The run-in phase with a free diet prior to the baseline visit served as a 4-week reference period, where participants acted as their own controls, and no differences were observed during that time. The improvement in WOMAC scores seen in our study was strongly correlated with a decrease in BMI, aligning with extensive literature supporting the efficacy of weight loss interventions in improving WOMAC outcomes [67].

Thus, while the level of WOMAC improvement achieved with VLCKD is similar to that reported in placebo-controlled trials, we believe this effect was not due to placebo, but rather a direct result of weight reduction [74]. Although our study design precluded the use of a placebo group, future research could explore the benefits of VLCKD compared to a standard low-fat diet to determine whether ketone bodies offer additional anti-inflammatory effects beyond those attributed to weight loss [25]. The ability to achieve substantial weight loss in a short period makes VLCKD a promising option for patients with obesity and symptomatic knee OA.

Finally, we recognize that longer-term studies are necessary to assess whether knee OA patients can maintain weight loss following VLCKD.

## 5. Conclusions

In conclusion, a 20-week VLCKD program in women with obesity and knee OA led to significant weight loss and improvements in all PROs, including WOMA, EQ-5D, and SF-36. The retention rate was high, and adverse events were minor and transient. Larger studies are needed to confirm these results and to determine the extent to which the benefits of VLCKD are attributable to weight loss alone or if the diet exerts anti-inflammatory effects. Nonetheless, the positive findings suggest that VLCKD could be an effective therapeutic option for patients with obesity and knee OA.

## Figures and Tables

**Table 1:** Eligibility criteria.

Inclusion Criteria
Age ≥ 18 years and ≤65 years
AND
Symptomatic knee osteoarthritis according to the American College of Rheumatology criteria [45]
AND
History of failure to achieve weight loss with standard hypocaloric diets
AND
Body mass index (BMI) ≥ 35.0 kg/m^2^
OR
BMI between 30.0 and 34.9 kg/m^2^ plus at least one additional cardiometabolic risk condition: ○ Past diagnosis of type 2 diabetes without β-cell failure; ○ Hypertriglyceridemia (fasting triglycerides ≥ 150 mg/dL) or taking lipid-lowering medications; ○ Hypercholesterolemia (total cholesterol > 200 mg/dL) or taking lipid-lowering medications; ○ Past diagnosis of arterial hypertension or taking blood pressure-lowering medications; ○ Past diagnosis of non-alcoholic fatty liver disease; ○ Past diagnosis of heart failure class NYHA I–II; ○ Past history of myocardial infarction or stroke/minor stroke (>12 months); ○ Past diagnosis of carotid atherosclerosis; ○ Past diagnosis of polycystic ovary syndrome (PCOS); ○ Past diagnosis of neurodegenerative disorders;
**Exclusion Criteria**
Current pregnancy or breastfeeding; Past diagnosis of type 1 diabetes mellitus or latent autoimmune diabetes in adults; Past diagnosis of type 2 diabetes without β-cell failure; Use of sodium/glucose cotransporter 2 (SGLT2) inhibitors; Past diagnosis of kidney failure and moderate-to-severe chronic kidney disease, liver failure, hearth failure NYHA III–IV, respiratory failure; Past diagnosis of unstable angina; Recent stroke or myocardial infarction (<12 months); Cardiac arrhythmias; Past diagnosis of eating disorders and other severe mental illnesses, alcohol and substance abuse; Active infections or severe chronic infections; Elective surgery or invasive procedures scheduled during the study period; Past diagnosis of rare disorders such as porphyria, carnitine deficiency, carnitine palmitoyltransferase deficiency, carnitine-acylcarnitine translocase deficiency, mitochondrial fatty acid β-oxidation disorders, pyruvate carboxylase deficiency; Allergy to protein preparation ingredients; Past or current history of gallstones.

**Table 2:** Baseline characteristics of patients who completed the study.

Characteristics	Patients (*n* = 16)
Age, years, mean ± SD	57.3 ± 5.5
BMI, kg/m^2^, mean ± SD	40.0 ± 4.8
BMI > 35, *n* (%)	13 (81)
Comorbidities	
Hypertension, *n* (%)	9 (56)
Hyperlipidemia, *n* (%)	8 (50)
Non-alcoholic fatty liver disease, *n* (%)	5 (31)
Polycystic ovary syndrome, *n* (%)	1 (6)
Type 2 diabetes, *n* (%)	1 (6)
Previous atherothrombotic events, *n* (%)	1 (6)
Congestive heart failure, *n* (%)	0
Neurodegenerative disorders, *n* (%)	0
Medications	
Non-steroidal anti-inflammatory drugs, *n* (%)	6 (38)
Paracetamol, *n* (%)	9 (56)
Patient-reported outcomes	
Total WOMAC, mean ± SD	43.6 ± 16.9
WOMAC pain, mean ± SD	8.0 ± 3.1
WOMAC stiffness, mean ± SD	4.2 ± 1.9
WOMAC function, mean ± SD	31.3 ± 13.0
EQ-5D utility score, mean ± SD	0.72 ± 0.32
EQ-5D VAS, mean ± SD	54.9 ± 24.0
SF-36 MCS, mean ± SD	53.6 ± 23.7
SF-36 PCS, mean ± SD	46.2 ± 24.8

BMI: body mass index; EQ-5D: EuroQoL 5 Dimensions; MCS: mental component score; PCS: physical component score; SD: standard deviation; SF-36: 36-item Short Form Health Survey; VAS: visual analog scale; WOMAC: Western Ontario and McMaster Universities Osteoarthritis Index.

**Table 3:** Assessment of urinary ketones.

Patient	Time Point of the Study									
	T0	T1	T2	T3	T4	T5	T6	T7	T8	T12
OA1		●	●	●●	●●	●		●		
OA3				●	●	●●	●		●	
OA4		●●	●●	●●●	●●●	●●	●			
OA5				●	●●	●	●●	●	●	●
OA6		●	●	●	●	●●	●●	●	●	
OA7		●	●●	●●●	●●	●●	●	●●	●	●
OA9		●	●	●●	●	●	●			
OA10		●	●●	●	●●	●●	●	●		
OA11		●	●	●	●●	●●	●●	●	●	●
OA12		●	●	●●	●●●	●●●	●	●	●	
OA14		●	●	●	●●	●	●●		●	
OA15			●	●	●●	●	●	●	●	
OA16		●●	●●●	●●●	●●	●●	●	●		
OA17		●	●	●	●	●	●	●	●	
OA19		●	●	●●	●	●	●			
OA20			●	●●	●●●	●	●	●		
Amount of ketones in the urine:			absent		● small		●● moderate		●●● large	

**Table 4:** Association of BMI and patient-reported outcomes over time.

	β Coefficient (95% CI)	*p*-Value
Outcome: total WOMAC index over time		
BMI	1.43 (0.29 to 2.58)	0.014
Outcome: WOMAC pain over time		
BMI	0.26 (−0.17 to 0.54)	0.066
Outcome: WOMAC stiffness over time		
BMI	0.12 (0.00 to 0.23)	0.044
Outcome: WOMAC function over time		
BMI	1.07 (0.26 to 1.89)	0.010
Outcome: EQ-5D utility score over time		
BMI	−0.01 (−0.17 to 0.00)	0.055
Outcome: EQ-5D VAS score over time		
BMI	−1.74 (−3.09 to −0.39)	0.012
Outcome: SF-36 MCS over time		
BMI	−1.35 (−3.15 to 0.45)	0.140
Outcome: SF-36 PCS score over time		
BMI	−3.25 (−4.65 to −1.84)	<0.001

BMI: body mass index; EQ-5D: EuroQoL 5 Dimensions; MCS: mental component score; PCS: physical component score; SF-36: 36-item Short Form Health Survey; VAS: visual analog scale; WOMAC: Western Ontario and McMaster Universities Osteoarthritis Index.

## Data Availability

The data presented in this study are available from the corresponding author upon reasonable request.
